# Cancer and changes in facial appearance: A meta‐ethnography of qualitative studies

**DOI:** 10.1111/bjhp.12398

**Published:** 2020-01-02

**Authors:** Andrew R. Thompson, Iona Sewards, Sarah R. Baker

**Affiliations:** ^1^ Clinical Psychology Unit Department of Psychology University of Sheffield UK; ^2^ School of Clinical Dentistry University of Sheffield UK; ^3^ Present address: Rotherham General Hospital UK

**Keywords:** cancer, coping, facial appearance, meta‐ethnography, stigma, visible difference

## Abstract

**Introduction:**

Living with an altered facial appearance as a result of treatment for cancer requires considerable psychological adjustment. As such it is essential that health care professionals understand the lived experience of people affected. This systematic review provides a meta‐ethnography of studies that have explored the experience of changed facial appearance as a result of cancer.

**Methods:**

A search of four databases (Web of Science, CINAHL, PsycInfo, and Scopus) took place using terms relating to qualitative research, cancer, and changed facial appearance. Thirteen studies were identified, appraised, and included in the synthesis. The findings and interpretations within the studies were subject to meta‐ethnography procedures so as to elicit novel cross‐cutting themes.

**Findings:**

The experience of changed facial appearance after cancer was clustered into three contexts. In the context of the disease, subthemes were the primacy of survival, the changing relationship with the disease, and the impact of the care team on the experience of changed appearance. In the context of the social world, subthemes were positive reactions, negative reactions, and coping strategies. In the context of the self, subthemes were the self under attack, self‐to‐self relating, the self in the world, and rebuilding the self.

**Conclusions:**

The findings indicate that health care professionals must conduct holistic assessments, so as to fully recognize and where necessary address the impact upon self. The meta‐ethnography shows that the experience of facial appearance change following cancer is complex and requires awareness of a number of theoretical areas including identity construction, social support, stigmatization, and the specific literature on visible difference.

**Statement of contribution**

***What is already known on this subject?***

- Changed facial appearance after cancer can cause significant social difficulties and impact on the sense of self.
- The experience of managing the specific dual challenges of cancer and altered facial appearance is not clearly understood.
- Recent studies that have focused specifically on the experience of changes in appearance after cancer have been limited in scope and transferability.

***What does this study add?***

- To our knowledge, this is the first meta‐ethnography to bring together the literature on the impact of altered facial appearance following cancer.
- Patients may feel unable to talk about appearance with health care professionals because it is seen as a frivolous issue.
- Clinicians should facilitate open, person‐centred opportunities for patients to discuss the impact of changed appearance and where necessary facilitate access to support.

## Background

Head and neck cancer (HNC) covers a range of cancers affecting the head and neck including oral, nasal, and orbital areas. Globally, there are 550,000 cases of HNC annually, and in some countries, the incidence is increasing (Bray *et al.*, 2018). Whilst survival from HNCs has become more commonplace, the disease and treatments often cause significant physical changes including marked disfigurement. HNC does not normally include skin cancers; however, skin cancer is often treated with resecting surgery that can change facial appearance in a similar way to HNC. Surgery around the neck can also result in constricted lymphatic flow in the face, leading to temporarily altered facial appearance (McGarvey, Osmotherly, Hoffman, & Chiarelli, 2014). Reconstructive surgery once the cancer has been removed can help restore appearance although patients are typically left with some level of permanent appearance change (Williamson & Wallace, 2012). Not surprisingly changes to facial appearance resulting from cancer or iatrogenic damage can have a significant impact on psychosocial adjustment (Rhoten, Murphy, & Ridner, 2013).

Adjustment to changes in facial appearance following cancer varies considerably with some studies having identified the presence of positive adjustment in this population as demonstrated by measures of quality of life, depression, and anxiety (Katz, Irish, Devins, Rodin, & Gullane, 2003; Vickery, Latchford, Hewison, Bellew, & Feber, 2003). Conversely, other studies report elevated levels of depression, social anxiety, poor body image, and shame (Clarke, Newell, Thompson, Harcourt, & Lindenmeyer, 2014; Fingeret *et al.*, 2012; Neilson *et al.* 2013). These equivocal findings may be a result in part of the varying range of methods employed by the different studies, although such findings are likely to suggest that adjustment is a product of a complex interaction between biomedical, demographic, and individual difference factors (Clarke *et al.*, 2014). Indeed, several studies indicate that women and those with low levels of social support are most at risk of psychological distress (Katz *et al.*, 2003; Bowers, 2008; Caddick *et al.*, 2012). Some studies also indicate a clear relationship between anatomic site of the cancer and mood disturbance such as depression. For example, Rohde *et al. *(2018) in a retrospective analysis of over 71,000 cases of HNC drawn from an American data set found that whilst the overall prevalence of depression was 9.3%, there was a wide variation in prevalence based on anatomic site, with laryngeal cancers being associated with the highest prevalence at 28.5%. This is perhaps not surprising as anatomic site will have a direct impact on functioning and thus quality of life (Terrell *et al.*, 2004).

Whilst demographic and biomedical factors may play a role in adjustment it is now acknowledged in the wider field of visible difference that psychosocial variables such as optimism and the extent to which appearance is valued or salient within the self‐concept play a crucial role (Thompson & Kent, 2001; Rumsey & Harcourt, 2005; Clarke *et al.*, 2013; Coneo, Thompson, Lavda, & The Appearance Research Collaboration, 2017; Moss, Lawson, White, & The appearance Research Collaboration, 2014).

Whilst the objective levels of adjustment and quality of life in cancer patients with altered facial appearance are well documented, the phenomenological experience of managing the specific dual challenges of cancer and altered facial appearance is less clearly understood (Williamson & Wallace, 2012). Qualitative research uniquely provides access to patients’ experiences in such a way that as to contribute to theory development by the identification of novel processes that might be implicated or be operating in adjustment (Thompson & Kent, 2001; Harper & Thompson 2011). A number of qualitative studies have been conducted with people who have survived head and neck cancer (HNC), and Lang, France, Williams, Humphris, and Wells, (2013 conducted a meta‐ethnographic review of 29 studies that had examined the experience of living with HNC. Lang *et al.* identified six core themes in the studies synthesized with concerns about appearance changes being present across several of these themes. The meta‐ethnography identified a loss of sense of self and social disruption as being a consequence of changed appearance for many people living with HNC. The Lang *et al.* review focused on HNC, and many of the included studies focused on the experience of physical adjustment. As such the Lang *et al.* review does not synthesize the literature in a way that enables nuanced consideration of the specific impact of changes to facial appearance following treatment for cancer.

There is an emerging literature that has focused on specific aspects of the experience of change in appearance following cancer. These qualitative studies have limited transferability in so far as they have largely focused on specific conditions or issues. Consequently, this review seeks to synthesize the individual studies, thus enabling transferability across a greater range of conditions and to the wider theoretical literature on visible difference. There are a number of approaches available for synthesizing qualitative studies with some aiming to summarize and group findings across studies, and others seeking to go beyond this and generate new interpretations based on the synthesized data (Toye *et al.*, 2014). Meta‐ethnography (ME: Noblit & Hare 1988) is a widely used and respected approach that enables generation of new interpretations from data synthesized from qualitative studies. ME has been posited to be particularly well suited to the generation of new knowledge when there is a relatively small set of studies available for inclusion in a review, as is this case with altered facial appearance following cancer (Campbell *et al.*, 2011). Consequently, a meta‐ethnographic approach was specifically employed within the current review, as the aim was to synthesize the findings of individual studies in such a way as to add to the theoretical understanding of altered facial appearance following cancer.

## Methods

The review was conducted in three stages as per the established ME approach (Noblit & Hare, 1988). First, a systematic search of qualitative studies looking at aspects of changed facial appearance as a result of cancer. Second, quality appraisal of the studies. Third, synthesis of the findings.

### Stage 1: Search

**Figure 1:**
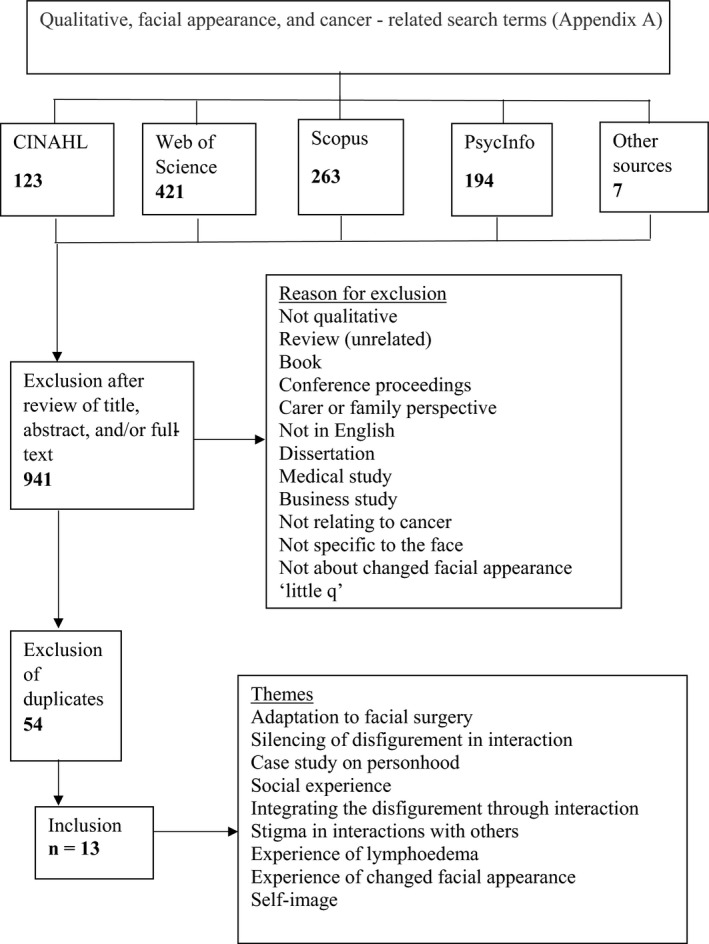
Process of selecting papers for inclusion.

Studies were included if they specifically aimed to investigate some aspect of the experience of changed facial appearance. Inclusion and exclusion criteria are specified in Table 1. A search was conducted using four electronic databases covering all available time points: Web of Science, CINAHL, PsycInfo, and Scopus. Citation and ancestry searches were conducted, which yielded several additional studies. Search terms were identified using the following: Sample, Phenomenon of Interest, Design, Evaluation, Research type (SPIDER) tool (Cooke, Smith, & Booth, 2012; Shaw, 2012) alongside keywords noted in the literature (Fingeret, Teo, & Goettsch, 2015; Lang *et al.*, 2013; Rhoten *et al.*, 2013). The list of search terms and further information on the search strategy deployed is documented in the Appendix S1 files. Figure 1 illustrates the selection process.

**Table 1:** Inclusion and exclusion criteria

Inclusion	Exclusion
Qualitative studies taking a ‘Big Q’ approach (i.e. reflexive analysis of rich data; Kidder & Fine, 1987)	Qualitative studies taking a ‘little q’ approach (i.e. qualitative data collection without reflexive analysis; Kidder & Fine, 1987)
Subjects: individuals with a past or present diagnosis of cancer affecting the head, neck, or face	Not relating to cancer Focus only on family or carer perspectives
Phenomenon of Interest: the experience of changed facial appearance resulting from cancer	Not relating to experience of changed facial appearance Studies of non‐facial appearance
Peer‐reviewed journal study article	Not in English

### Stage 2: Quality appraisal

All studies were appraised using a tool (see Appendix S1) based on the Critical Appraisal Skills Programme (CASP) Checklist for Qualitative Research (CASP, 2018), and a quality framework developed by the National Centre for Social Research (National Centre for Social Research, 2003). These modified tools have been used in previous meta‐ethnographic studies (Campbell *et al.*, 2003; Malpass *et al.*, 2009). The level of quality of each study was classified (see Appendix S1) according to set categories (Dixon‐Woods *et al.*, 2007).

All studies were appraised for quality by the first and second authors and 20% (*n* = 3) were selected at random and appraised by the third author. No discrepancies were reported in rating between the first and second authors. Discrepancies in the appraisal were noted for one item in two of the studies between the ratings of first, second, and third authors, and agreement was reached through discussion (see Appendix S1). Regardless of quality, all of the studies identified in the systematic search were included in the meta‐ethnography so as to encompass the full range of potential themes. However, quality concerns are discussed alongside the results to determine the contributions of each study and to present the methodological issues evident in the field.

### Stage 3: Synthesis of studies

Meta‐ethnography is the interpretative, rather than integrative, synthesis process (Harper, & Thompson, 2011) and aims to identify novel themes. Accordingly, studies in the present review were synthesized based on their first‐, second‐, and third‐order constructs (Table 2) over four phases as used by Malpass *et al.* (2009). The process of analysis was led by the second author and audited by the first author. The audit process involved review of all of the ‘data’ extracted at each phase and checking the records made back to the original studies.

1. *Phase 1: *All studies were read and reread in chronological order, noting the second‐order constructs identified by the original authors and patterns in themes across studies.
2. *Phase 2: *A table of second‐order constructs was created with reference to raw data in the original studies (first‐order constructs). To aid the interpretative process, a conceptual map was drawn for each study to link second‐order constructs with the original authors’ thereby maintaining the contextual meaning of each study (see Appendix S1 for an example).
3. *Phase 3: *Constructs from each study were translated into each other by comparing the themes across studies. Second‐order constructs were analysed for latent meaning.
4. *Phase 4: *Third‐order constructs were generated based on Noblit and Hare’s (1988) method of generating ‘lines of argument’. To achieve this, translated constructs were grouped into new conceptual contexts.

**Table 2:** First‐, second‐, and third‐order constructs

First‐order construct	*Interpretations of experience:* the participant’s account of having changed facial appearance as a result of cancer
Second‐order constructs	*Interpretations of interpretations of experience:* the themes developed by the original authors
Third‐order constructs	*Interpretations of interpretations of interpretations of experience:* the new concepts and themes developed in the process of synthesis

## Results

Two articles were identified from the same study: Bonanno and Choi (2010) looked at individual experiences of HNC patients in social interactions, whilst Bonanno and Esmaeli (2012) used the same data to explore factors around group size. Both articles were included in the synthesis in the same way, with acknowledgement of repeated themes arising.

The studies covered different aspects of the unique experiences of individuals from a wide range of contexts. Most studies focused on HNC or facial cancer surgery; however, some studies looked at multiple cancers (Speraw, 2009), skin cancer (Lee *et al.*, 2016), and lymphoedema (McGarvey *et al.*, 2014). Participants in the studies were 58% female (*n* = 118) and covered a wide range of ages (16–84 years). Most participants were based in North America or Europe; however, participants from Brazil and India were represented in two studies. All studies used semi‐structured interviews with individuals as the primary means of data collection, followed by line‐by‐line coding of qualitative data to identify themes. The methodological approach used was not specified in three studies; all other studies used either grounded theory (GT) or a phenomenological approach (PA). Table 3 summarizes each article included in this review.

**Table 3:** Summary of details and main themes of selected studies

	Author (year)	Setting and country	Participants	Aim	Design	Analysis method	Key themes	Quality appraisal
1	Furness *et al.* (2006)	Community and outpatient, UK	Facial surgery patients *n* = 29 (cancer patients *n* = 21). 65% female, 35% male. Age 34–84	Explore and explain the experience of adapting to facial surgery	Semi‐structured focus group; individual interviews	Grounded Theory	Demands, resources, responding and managing, and consequences of facial surgery	SAT
2	Konradsen *et al.* (2009)	Inpatient, Denmark	Facial cancer surgery patients. *n* = 14. Female = 5, male = 9. Age 25–78	Explore and explain changed facial appearance and nurse–patient interactions following cancer surgery	Semi‐structured interviews, everyday nurse–patient interactions	Grounded Theory	Minimizing appearance, appearance is a luxurious problem and another time, another place	KP
3	Speraw (2009)	Community, USA	1 female with multiple facial cancer treatments, age 16	Explore the concept of personhood in a case study	Semi‐structured individual interview	Thomas & Pollio’s (2002) phenomenological approach	Personhood and agency in health care	SAT
4	Turpin *et al.* (2009)	Outpatient, UK	HNC surgery patients with altered appearance, *n* = 10. Male = 6, female = 4. Age 41–66	Explore the personal meaning and impact of HNC, in particular the individual’s sense of self	Semi‐structured interviews and Repertory Grids	Interpretative Phenomenological Analysis	Destruction of self, altered relations to body, disenfranchised self, and conservation of self	KP
5	Van Doorne, van Waas, and Bergsma (1994)	Outpatient and community, The Netherlands	HNC patients with changed facial appearance, *n* = 24. Male = 19, female = 5. Average age 65	Explore coping in cancer patients with changed facial appearance	Reflective, semi‐ structured individual interviews	Unspecified	Fear of dying, appearance changes as the price for survival, coping	Q
6	Bonanno and Choi (2010)	Cancer hospital, USA	HNC surgery patients, *n* = 14. Male = 8, female = 6. Age 31–81	Analyse patterns of social interaction as experienced by people with changed facial appearance after cancer	Semi‐structured phone interviews with individuals and family members	Grounded Theory	Intrusion, sympathy, and benign neglect in social interactions for individuals who are always or only occasionally comfortable	Q
7	Konradsen *et al.* (2012)	Community and outpatient, Denmark	Facial cancer surgery patients, *n* = 12, Female = 6, male = 6. Age unspecified	Understand the ongoing process of adjustment to changed facial appearance	Semi‐structured individual interviews	Grounded Theory	Interactional integration of changed appearance facilitates the progression of adjustment	SAT
8	Bonanno & Esmaeli (2012)	Cancer hospital, USA	HNC surgery patients, *n* = 14. Male = 8, female = 6. Age 31–81	Analyse patterns of social interaction for people with changed appearance after HNC	Semi‐structured interviews with individuals and family	Grounded Theory	Intrusion, sympathy, and benign neglect in social interactions in large or small groups	SAT
9	McGarvey *et al.* (2014)	Outpatient, UK	HNC patients with lymphoedema, *n* = 10. 80% male, 20% female. Age 32–75	Explore how lymphoedema following HNC treatment affects patients	Semi‐structured individual interviews	Unspecified	Negative psychosocial sequelae of lymphoedema, coping strategies	SAT
10	Costa, Nogueira, Lima, Mendonca, and Leles (2014)	Inpatient and outpatient, Brazil	Facial cancer surgery patients, *n* = 10. Male = 5, female = 5. Age 26–72	Explore experience of changed facial appearance after HNC surgery	Semi‐structured individual interviews	Grounded Theory	Discovering cancer, coping with cancer and appearance, reconstructing a new identity	SAT
11	Henry *et al. *(2014)	Outpatient, Canada	HNC surgery patients with changed facial appearance, *n* = 14. Male = 7, female = 7. Age 39–79	Explore the lived experience of changed facial appearance in the course of HNC	Semi‐structured individual interviews	Interpretative Phenomenological Analysis	Oscillations between ruptured self‐image and normalcy and acceptance	KP
12	Nayak *et al.* (2016)	Tertiary care, India	7 female HNC patients. Age unspecified	Understand self‐image in HNC patients	Semi‐structured individual interviews	Colaizzi’s (1978) approach	Valuing the internal above the external self	FF
13	Lee, Klassen, Lawson, Cano, Scott, and Pusic (2016)	Cancer hospital, USA	Facial skin cancer surgery patients, *n* = 15. Male = 6, female = 9	Identify aesthetic and health issues of facial cancer surgery patients	Semi‐structured individual interviews	Unspecified; line‐by‐line coding and constant comparison	Appearance‐related, psychological, social and physical concerns, and satisfaction with care	SAT
			3 aged 20–40, 5 aged 40–60, 7 aged 60–80					

The synthesis process identified 10 third‐order constructs synthesized from 45 constructs (Table 4). Third‐order constructs were grouped into three conceptual categories. First, the ‘context of the disease’: this included the primary importance of survival which puts appearance into perspective, the relationship with the disease and the disease’s impact on experiences of appearance over the course of the cancer journey, and experiences with the care team. Second, the ‘context of the social world’: this included positive and negative reactions, and the strategies used to cope with these. Third, the ‘context of the self’: this included the attack to the self, resulting from the changed appearance, self‐to‐self relating, the self in the world, and rebuilding a sense of self. Extracts from participant accounts are included as evidence of themes. Extracts were selected from all articles used in the review on the basis of being representative of the emerging theme.

**Table 4:** Themes supported by each article in the meta‐ethnography

Author (year)	Context of disease			Context of social world			Context of self			
	Survival is paramount	Relationship with disease	The care team	Positive reactions	Negative reactions	Coping strategies	Self under attack	Self‐to‐self relating	The self in the world	Rebuilding the self
Furness *et al. *(2006)	X				X	X	X			X
Konradsen *et al. *(2009)	X	X	X		X					
Speraw (2009)			X		X	X		X		X
Turpin *et al. *(2009)	X			X	X	X	X	X	X	X
Van Doorne *et al.*(1994)	X	X		X	X		X			X
Bonanno and Choi (2010)				X	X	X			X	
Konradsen *et al. *(2012)			X		X	X		X	X	
Bonanno and Esmaeli (2012)				X	X	X			X	
McGarvey *et al. *(2014)						X	X	X		
Costa *et al. *(2014)	X	X			X	X		X		X
Henry *et al. *(2014)	X	X	X	X	X	X	X	X	X	X
Nayak *et al. *(2016)	X					X				X
Lee *et al. *(2016)	X	X	X			X	X			

### The context of the disease

Several third‐order constructs related to participants’ experiences of their changed facial appearance. These often linked with the early stages of cancer and treatment, including the pre‐operative changes in appearance as symptoms became more apparent, and the post‐operative period when the extent of changed facial appearance was first evident after surgery.

#### Survival is paramount

A recurring theme in many studies (Costa *et al.*, 2014; Furness, Garrud, Faulder, & Swift, 2006; Henry *et al.*, 2014; Konradsen, Kirkevold, & Zoffmann, 2009; Turpin, Dallos, Owen, & Thomas, 2009; Lee *et al.*, 2016; Nayak, Pai, & George, 2016; Van Doorne *et al.*, 1994) was the importance placed on survival, and large changes to facial appearance were accepted in the context of having survived the disease, and as such were perceived as a necessary ‘trade‐off’ or price that needed to be paid for continued life:

‘I actually don’t give a toss what I look like because I’m alive, and I just think the issue of cancer returning and doing its worst, it’s a far bigger issue than how you look’ (Furness *et al.*, 2006).

Konradsen *et al. *(2009) found that participants’ experience of appearance as a luxury issue stemmed from interactions with nursing staff in the post‐operative period. Participants experienced a ‘silencing’ of their changed appearance in that body image was not discussed, and staff were all accustomed to seeing faces changed by surgery. In these early days after surgery, participants felt that they were lucky to be alive and therefore should not complain about their appearance:

‘So I feel that it’s kind of a luxury problem, and nothing to bother anyone with, the fact that I feel sad about looking this way’ (Konradsen *et al.*, 2009).

Costa *et al.*’s (2014) and Nayak *et al.*’s (2016) studies both found appearance changes were perceived as the ‘price’ for removing disease threat and that changed facial appearance was experienced fatalistically:

‘What do I have with beauty? If God gives good health, that’s enough’ (Nayak *et al.*, 2016)

#### Relationship with the disease

Participants’ relationships with the cancer and treatments varied over time, and this influenced how appearance changes were experienced. Most studies described an emotional journey from concern or denial when symptoms started, shock at the diagnosis, fear about the operation, disgust reactions to post‐operative facial appearance, relief that the threat of cancer has been dealt with, and worry about recurrence of the disease:

‘The first moment it really stunned me. The first moment is that shock…and you feel like: how can I live with this?’ (Costa *et al.*, 2014).

Once the cancer was successfully removed and the threat of dying was no longer at the forefront, participants were then faced with the task of living with a changed face, which consequently took on greater significance (Furness *et al.*, 2006). Later, when the physical wounds of the surgery had healed, changed facial appearance could act as an ever‐present reminder of the cancer, perpetuating feelings of worry about recurrence. Thus, the fear of dying was, for some, maintained by continuing to check for changes in facial appearance:

‘…I started looking to see if it was a bit red. And then you start wondering, “OK, so this is how the first one started….”’ (Henry *et al.*, 2014).

Social and practical problems meant that participants were constantly aware of their appearance and thus remained of the disease. The reactions of others drew participants’ attention back to the distressing narrative of cancer:

‘They’re talking about your neck, your scars. You hear different things… and part of you just feels like turning around and saying, ‘It’s cancer!’ (Henry *et al.*, 2014).

Facial appearance was also inextricably bound up with practical problems (Lee *et al.*, 2016), such as functional difficulties with eating or speaking, the need to clean the site, and sometimes the use of prostheses. The need to engage in healthy behaviours, for example reducing sun exposure, became a practical consideration.

‘This nose thing has changed my whole perspective. I now wear hats. I have sun block in my car’ (Lee *et al.*, 2016).

#### The care team

During the diagnosis and treatment period, the way in which the care team dealt with the changed facial appearance was important (Henry *et al.*, 2014; Konradsen *et al.*, 2009; Lee *et al.*, 2016; Speraw, 2009). Henry *et al. *(2014) reported that professionals who took an empathic, person‐centred, and open approach facilitated better adjustment to changed facial appearance;

‘The backup from the nursing staff is instrumental in making people happy’ (Lee *et al.*, 2016).

‘It’s so personalised that already it takes away the fear … in moments like these, that’s what you need the most – that little extra human touch’ (Henry *et al.*, 2014).

Participants’ descriptions suggest a balance is needed between acknowledging appearance changes without stigmatizing. Konradsen *et al. *(2009) found that appearance was minimized by the care team, meaning that the participant’s negative feelings about their appearance were silenced as they learned that altered appearance, whilst an important issue to them, was not something that was of importance to staff. Konradsen *et al. *(2012) found that acceptance of appearance changes only began once this silence was broken in interactions after the participant had left hospital. Speraw’s case study (2009) of a teenage girl with multiple cancers of the face found that she experienced considerable stigmatization by health care professionals in so much as nurses appeared to make incorrect assumptions about her abilities:

‘Just because my eyes are plastic and my ears are rubber doesn’t mean I shouldn’t be talked to like anyone else…I have the nurses yell at me ‘cos they act like I can’t hear at all’ (Speraw, 2009).

### The context of the social world

A number of third‐order constructs emerged relating to participants’ experiences of their altered facial appearance within a social context. These included the positive and negative reactions elicited by others, and the coping strategies employed by participants to manage in the social world.

#### Others’ positive reactions

Positive interactions during the treatment phase included empathic listening and explaining which helped participants to feel safe and accepted (Henry *et al.*, 2014). Beyond the hospital environment, participants described how explicit commenting on facial appearance, initiated by other people, could help them to feel ‘normal’, and enable them to begin integrating the changed appearance into a new self‐construct (Konradsen *et al.*, 2012). Having permission from others to have a forthright conversation about appearance could reduce the sense of ‘otherness’ by assuaging curiosity and concern which could otherwise engender stigmatization:

‘I went to my daughter’s school and told her classmates what had happened… Many children and their parents came up to me and asked … “why is your face swollen? Did anything happen to you?” and so on. So from that day on, everything became normal.’ (Konradsen *et al.*, 2012)

In contrast, sometimes the experience was more positive when appearance was not acknowledged at all. One study (Bonanno, & Esmaeli, 2012) found that stigma was not perceived when strangers or acquaintances behaved with a ‘benign neglect’. In group settings, when appearance was paid no special attention, participants felt ‘normal’ and enabled to be part of the group as they would have done before the cancer:

‘They just come and talk to me like I was totally normal…. when people treat you as you are totally normal, and I feel that way, it makes you feel good’ (Bonanno, & Esmaeli, 2012).

The number of people involved in an interaction also helped to determine how a response was perceived, for example sympathy and pity were more likely to be experienced as supportive when expressed in smaller groups (Bonanno, & Choi, 2010). In close relationships, comfort, support, and acceptance of changed appearance were highly valued across several studies as they enabled participants to talk about their appearance (Furness *et al.*, 2006; Van Doorne *et al.*, 1994).

*‘We talk about it, and it calms me down’* (A participant & her husband) (Furness *et al.*, 2006).

These interactions gave validation to participants’ concerns and reassurance that valued relationships would not be damaged by the changes in their appearance and gave them confidence that they could face the future in the context of these supportive relationships:

‘…the people who I most admire – my husband and my family – were supporting me…and I said, “these are the main things…then I will tackle the rest!”’ (Costa *et al.*, 2014).

Consistency and predictability in others’ reactions was a positive feature. When participants could predict how someone would react to their appearance, they felt more confident in interactions. However, unpredictable responses led to increased anxiety. For example, van Doorne *et al. *(1994) found that encountering partially known acquaintances was particularly problematic as responses were hard to predict. Complete strangers and close relations, however, tended to give consistent responses which reduced participants’ anxiety by enabling a degree of preparation.

#### Others’ negative reactions

Negative reactions from others in response to changed facial appearance were a significant theme in the majority of studies (Bonanno, & Choi, 2010; Bonanno, & Esmaeli, 2012; Costa *et al.*, 2014; Henry *et al.*, 2014; Konradsen *et al.*, 2012; Speraw, 2009; Turpin *et al.*, 2009; Van Doorne *et al.*, 1994). These included those of commission (e.g. intrusive questions or insults) and those of omission (e.g. awkward silences or being avoided). The most frequent negative reaction was an incongruent silence. Unusual silence in response to changed facial appearance was perceived as rejection, especially when from a close family member or friend. Some participants tried to talk about their appearance in close relationships but were met with silence and awkwardness which reinforced feelings of isolation (Furness *et al.*, 2006). Many participants described feeling like their appearance would make others uncomfortable and had a sense of responsibility for not putting upon others by exposing them to their changed faces (Turpin *et al.*, 2009):

‘Sometimes when you meet people, they are afraid to say anything because they are afraid of hurting you…that is a bit sad’ (Konradsen *et al.*, 2012).

Participants experienced provoking pity, sadness, and sympathy in others (Costa *et al.*, 2014; Henry *et al.*, 2014). Sometimes this was overbearing and out of proportion to participants’ perception of the severity of their changed appearance, which increased feelings of being inferior and different to others:

‘People treat me differently…they are sympathetic, overtly so… this is a person with a visual problem, we’ll treat him differently and they do … I think they feel sorry for me’ (Bonanno, & Choi, 2010).

Similarly, participants’ facial appearance prompted reactions of shock and disgust (Costa *et al.*, 2014; Henry *et al.*, 2014). People would stare, make comments, and ask questions. Bonanno and Choi (2010) describe how this felt intrusive and ranged from outright rudeness (‘she doesn’t have an eye! Look, look!’) to subtle looks (‘some of the mothers were looking at me strangely’). Such responses from others made participants uncomfortable and put pressure on them to share their personal stories with relative strangers, reinforcing feelings of being ‘abnormal’.

Sometimes participants reported being avoided by others. Sexual and intimate relationships became more difficult, with some participants experiencing outright rejection from those closest which clearly posed a major threat to self‐esteem:

‘He’s left me. He couldn’t cope with the way the disease had affected me … Now I have to go out and find a new boyfriend. That’s almost impossible with one eye’ (Konradsen *et al.*, 2012).

#### Coping strategies

Participants employed various strategies for coping with the demands placed on them by others’ reactions. Many studies identified social avoidance as a coping strategy (Costa *et al.*, 2014; Henry *et al.*, 2014; Konradsen *et al.*, 2012; McGarvey *et al.*, 2014). Participants expected to feel upset in social situations because of negative reactions, and so they avoided these situations (Konradsen *et al.*, 2012). This could lead to increased social isolation and perpetuated feelings of rejection:

‘I will stay at home; I hate being the centre of attention’ (Konradsen *et al.*, 2012)

Participants described how using concealment and camouflage helped them to appear and feel more ‘normal’, thereby reducing negative reactions (Costa *et al.*, 2014; Henry *et al.*, 2014; Lee *et al.*, 2016; McGarvey *et al.*, 2014; Turpin *et al.*, 2009). Participants used dark glasses, scarves, prostheses, long hair, and different postures to reduce the visibility of their facial appearance. Several participants described how they would not leave the house without using concealment.

Some participants valued religion as a coping strategy that could help them feel comforted and hopeful about their future with a different facial appearance (Costa *et al.*, 2014):

‘I went to the church… because if not I think I’d sink into depression and other consequences would come. Then I clung to God too…God has been my support… because if not, I guess I could not bear it’ (Costa *et al.*, 2014).

### The context of the self

Changed facial appearance had a significant influence on participants’ sense of themselves. In different studies, this concept was referred to as sense of self, self‐image, identity, and personhood. The experiences of changed facial appearance within the context of the disease and the context of the social world fed into the context of the self, so there is considerable conceptual overlap between themes.

#### Self under attack

The link between facial appearance and sense of self was apparent, as participants described physical changes in terms of an experience of loss of their inherent sense of self. Thus, participant’s concept of their personal identities was ruptured by changed facial appearance:

‘I felt that [the facial changes] took away from me, from my personality’ (Henry *et al.*, 2014).

Turpin *et al.* (2009) focused on the impact of HNC on the sense of self and found that the treatment phase of the disease marked a discontinuation from the previous self to the beginning of a new self:

‘You just can’t live the same life anymore; really you’re not the same person at all’ (Turpin *et al.* 2009).

Facial changes represented a loss of unique, self‐defining characteristics. The face had represented an essential part of an individual’s construction of themselves and changes to the face caused a rupture in this construction. Participants experienced the loss of valued social and occupational roles as a result of the disease and changed facial appearance, which was a further attack on the self as unique and valued.

‘My management style is persuasive … but I like to give the impression of a hardness … and pull back from that to ease the situation for whomever I might be managing … I am not able to do that now … so I’m disappointed and concerned that I’ve lost the ability to express myself properly’ (Turpin *et al.*, 2009).

#### Self‐to‐self relating

Across many studies, participants reported experiencing self‐critical appearance‐related thoughts (Costa *et al.*, 2014; Henry *et al.*, 2014; McGarvey *et al.*, 2014; Turpin *et al.*, 2009). They described themselves in emotive language as ugly, unattractive, undesirable, gross, and other terms linked to a sense of self‐disgust or shame:

‘My general appearance has been altered quite considerably…and all of a sudden to have a big bulbous neck like a cane toad’ (McGarvey *et al.*, 2014).

Numerous participants were reluctant to look at themselves in the mirror. Some participants would refer to themselves as subhuman, using terms like ‘alien’, ‘not human’, ‘circus beast’, and ‘mutant’. For these participants, the destruction of the face seemed to correspond with the destruction of what made them human:

‘Have you seen the film The Fly, well you don’t want to …this chap ends up …half man and half fly…and it’s not a very nice sight and I tended to feel I was a bit like that’ (Turpin *et al.*, 2009).

Participants felt disenfranchised from themselves, believing that their post‐cancer appearance did not match their true identity – the person they saw in the mirror was ‘not me’. Where the pre‐cancer face represented the authentic self, the changed face was a diminished, inadequate, and false version (Turpin *et al.*, 2009).

#### Self in the world

This theme considers the individual’s internal perception of themselves around other people. This differs from the external social experience discussed in the context of the social world, although the two are inevitably linked.

Participants were keenly aware of being different from others, of belonging to a separate category because of their appearance. Thus, many participants felt conspicuous around others and evaluated themselves negatively as a result of their perceptions of others reactions (Bonanno & Choi 2010; Bonanno, & Esmaeli, 2012; Henry *et al.*, 2014; Konradsen *et al.*, 2012; Turpin *et al.*, 2009).

‘I feel odd; I feel like people are staring at it’ (Lee *et al.*, 2016).

‘The look of my face is the elephant in the room’ (McGarvey *et al.*, 2014).

Whilst others’ reactions fostered this feeling, participants were inherently aware that their altered appearance would garner unwanted attention:

‘It’s just a bid red square, which almost has a bullseye with arrows pointing to it’ (Henry *et al.*, 2014).

As a result of negative self‐perceptions in the social world and a fear of unwanted attention, participants reported a pull towards social avoidance:

‘In my case, right in the face…I was afraid of being rejected in society, Thus in the first moment I wanted to hide’ (Costa *et al.*, 2014).

Participants also felt judged by others and inferior compared with society’s standards of beauty and ‘normality’ (Van Doorne, et al., 1994; Henry *et al.*, 2014).

#### Rebuilding the self

An adjustment process in which the sense of self was rebuilt over time was evident (Turpin *et al.*, 2009). This process was gradual and not straightforward; oscillations between old and new identities were common (Van Doorne et al., 1994; Henry *et al.*, 2014).

Whilst a desire to return to the ‘old’ self was often present, participants also strived to build a new identity in which they took pride in their ‘survivor’ status and could use their experiences for personal development (Costa *et al.*, 2014; Henry *et al.*, 2014). Participants continued to look back to valued aspects of their previous selves and hoped to continue their rehabilitation towards a better future.

Several factors influenced the process of rebuilding the self. Some participants felt that their older age and later life stage protected them to some extent (Lee *et al.*, 2016; Nayak *et al.*, 2016; Turpin *et al.*, 2009):

‘Not that at the end of the day it really matters, because I’m not a single young man who’s likely to be just setting out in life’ (Turpin *et al.*, 2009).

Gender also played a role, with female participants in the available sample more concerned with appearance changes (McGarvey *et al.*, 2014) and men being more concerned with functional problems (Henry *et al.*, 2014).

## Discussion

This study reveals that facial appearance changes stemming from cancer can have a profound impact on individuals’ sense of identity, for some even triggering a state of feeling as if they are no longer themselves. For many others, strong thoughts of self‐rejection are associated with feelings of shame and self‐disgust. Many people recreate themselves as survivors yet they have to learn how to manage both their own and others often continuing burdensome negative reactions towards their altered appearance. Support and acceptance from others appear to be crucial in the adjustment process.

The findings of this review are largely in line with previous research into changed facial appearance (Cooke Macgregor, 1990; Lebel, Castonguay, Mackness, Irish, Bezjak, & Devins, 2013; Thompson & Kent, 2001). However, the findings therefore extend those of Lang *et al. *(2013); in particular, Lang *et al.*’s theme of ‘the diminished self’ is supported, but the present review clarified that appearance concerns are as significant a threat to self‐identity as functional difficulties.

Unfortunately, the synthesized studies appear to have largely captured the negative aspects of the experience, and as such Goffman’s concept of stigma (Goffman, 1963) is particularly relevant to understanding the extant findings. Scambler & Hopkins (1986) elaborated Goffman’s concept into ‘felt’ and ‘enacted’ stigma, which are predominantly concerned with internal judgements/cognitions as to whether stigmatization has occurred and is justified. Other theorists have focused on the affective states that might accompany such perceptions. For example, the concept of feelings of internal and external shame has been explicitly linked to concerns about social connectedness (Lewis, 1992; Kent & Thompson, 2002). Internal shame and a fear of rejection or judgement from others were encompassed in the findings in this review within the contexts of ‘self under attack’, ‘self‐to‐self relating’, and the ‘self in the world’. A loss of identity, loss of roles, negative body image, loss of personhood, self‐consciousness, and perceived inferiority compared to society’s standards of ‘normal’ appearance are aspects of felt stigma included here that have been reported in other studies and reviews (Callahan, 2005; Thompson, & Kent, 2001). Enacted stigma refers to awareness of discriminatory behaviours of others, and for the participants whose data were included in this review, reports of experiences of enacted stigma ranged from awkward silences through to insults and outright rejection in some cases in close relationships.

Several theorists have posited that the emotion of shame might be closely associated with the phenomenon of stigmatization (Kent & Thompson, 2002; Thompson & Kent, 2001). A distinction has been made between internal and external shame, and this has also been discussed in the specific context of body related shame (Gilbert & Miles, 2002; Lewis, 1992). Internal shame stems from self‐focused criticism of the self or body as undesirable, whereas external shame is a feeling associated with awareness that others see the self as undesirable or unacceptable in some way. Theories of shame and stigmatization are particularly relevant to the findings of this review as facial difference is particularly conspicuous in social interaction, and cancer is often (and on occasion mistakenly) linked with judgements associated with lifestyle choices such as smoking and alcohol use (Ragin, Modugno, & Gollin, 2007).

Previous reviews have called for greater consideration on exploring potential positive aspects of the adjustment process following acquired disfigurement (Rumsey, & Harcourt, 2005; Thompson & Kent, 2001), and the extant studies included in this review indicate that there are some positive factors associated with the experience of head and neck cancer. As in the studies included in this review, Egan, Harcourt, Rumsey, Appearance Research Collaboration, & McBain, (2011) qualitative study found that receiving positive reactions from others, acceptance in close relationships, and seeing the difference as a means of personal growth are central to good adjustment. Other studies that have purposively investigated positive adjustment strongly suggest that people with a noticeable difference may continue to feel anger and humiliation in response to the intrusive reactions of others despite reporting having learnt strategies for managing such reactions (Thompson & Broom, 2009). As found in other studies (Karnell, Christensen, Rosenthal, Magnuson, & Funk, 2007; Katz *et al.*, 2003), this review indicates that social support plays a vital role to positive adjustment and this is unsurprising given the sense of acceptance that such support is likely to convey.

Whilst the review has added to knowledge of the importance of facial appearance in the experience of cancer, there were a number of limitations in the literature included, which may pose a threat to the dependability of the themes generated by the meta‐ethnography. Three of the studies included were of questionable quality (Bonanno & Choi, 2010; Van Doorne *et al.*, 1994; Nayak *et al.*, 2016). Van Doorne *et al.*’s (1994) study did not substantiate findings with participant quotes meaning that themes are difficult to confirm from the participants’ perspective. The dependability of the findings was also compromised by a lack of methodological detail (the recruitment strategy was not specified, and there was no indication of how themes were drawn from the data). Whilst Bonanno and Choi’s (2010) study provided sufficient rationale and detail for recruitment and data collection, it contained limited information on the method of analysis deployed, thus limiting the trustworthiness of the findings. Nayak *et al.*’s (2016) study was classified as being ‘fatally flawed’ in quality as it did not describe how participants were approached, how the semi‐structured interviews were conducted, or how the data were analysed. Further, there was insufficient detail provided of the participants’ characteristics, and the discussion of the findings was superficial. Participant quotes were provided to support themes; however, regardless of this given the methodological issues the findings of this study need to be interpreted with caution.

Some of the studies included in the meta‐ethnography were of questionable quality; however, the risk of these studies overly influencing the findings was mitigated against by the findings of two studies that were judged as being high quality being seen as being of particular importance when carrying out the analysis (Konradsen *et al.*, 2009; Turpin et al., 2009). Turpin *et al. *(2009) thoroughly described the procedures used throughout the research, particularly in relation to the analytic approach and procedures. Furthermore, the aim of the study, to explore sense of self in HNC patients, was highly relevant to the current review. Konradsen *et al. *(2009) aimed to explore interactions for those with changed facial appearance after cancer which was also invaluable to this review. A stepwise description of the GT procedures used at recruitment, data collection and analysis gave this study high credibility and transferability, and participant quotes alongside in‐depth contextual commentary added to dependability. The remaining studies were of satisfactory quality.

Whilst the meta‐ethnography followed accepted procedures, it may have been subject to some bias during study selection as this procedure was largely carried out by the second author. In addition, whilst the authors were mindful of the role that might be played by their existing theoretical knowledge and sought to minimize any bias that this might introduce via discussion between authors; the themes generated by the meta‐ethnography may nevertheless have still been influenced by these existing concepts.

This review has implications for health care professionals working with people with changed facial appearance after cancer. HNC patients may feel unable to talk about appearance with health care professionals because it is seen as a ‘luxury’ or frivolous issue. Therefore, medical and nursing care teams should acknowledge the potential psychosocial impact of appearance and create opportunities for conversation, whilst being mindful of behaviours which may be perceived as stigmatizing. This study suggests that social support, especially opportunities for experiencing positive reactions from others and being accepted regardless of changes in appearance, is central to positive adjustment. Receiving peer support from people who have experienced similar changes maybe particularly valuable in building a sense of community that can facilitate building of self‐esteem, and health care professionals might look to more readily sign post patients to organizations such as Changing Faces (see http://www.changingfaces.org.uk). Indeed, a recent study with HNC patients and carers (Al Gtewi, Owens, & Baker, 2017) found evidence to suggest that the use of online support groups may be associated with better quality of life and lower levels of depression and anxiety.

The findings from this review indicate that the current psychological interventions available for people post HNC may be too focussed on targeting individual cognitive factors, and our findings suggest that greater consideration is needed within interventions of assisting people to manage the reactions of others whilst at the same time developing self‐compassion. Targeted forms of cognitive behavioural therapy (e.g. Clarke *et al.*, 2013) and approaches such as Acceptance and Commitment Therapy (Feros, Lane, Ciarrochi, & Blackledge, 2013; Shepherd, Reynolds, Turner, O'Boyle, & Thompson, 2019; Zucchelli, Donnelly, Williamson, & Hooper, 2018) and Compassion‐Based Therapy (Kirby, Tellegen, & Steindl, 2017; Hudson *et al.* in second review) might be particularly useful to facilitating adjustment for people coming to terms with living with an altered appearance.

Finally, whilst there is some research (Llewellyn, Horney, McGurk, Weinman, Herold, Altman, & Smith, 2013; Thambyrajah, Herold, Altman, & Llewellyn, 2010) already addresses the role of benefit finding and positive adjustment in cancer, but future research is needed to investigate this in the context of cancers affecting facial appearance. Given the importance of time in the adjustment to cancer, longitudinal qualitative research is needed to increase our understanding of how people learn to manage both their own and others reactions.

## Supporting information


**Appendix S1.** File containing further detail of the methods used in the meta‐ethnography.
