# Supplementary material for: Cancer and changes in facial appearance: A meta‐ethnography of qualitative studies
Source: Br J Health Psychol. 2020 Jan 2;25(1):129–51. doi: 10.1111/bjhp.12398 (PMC7003786; doi:10.1111/bjhp.12398)
Supplement: Supplementary file 1 — Appendix S1. File containing further detail of the methods used in the meta‐ethnography. [file BJHP-25-129-s001.docx]

**Supporting information**

**Literature review search strategy**

In the first instance, titles and abstracts of all articles were screened to assess relevance to the review question according to the inclusion (qualitative studies taking a ‘Big Q’ approach; individuals with past or present diagnosis of cancer affecting the head, neck or face; the experience of changed facial appearance resulting from cancer; peer-reviewed journal article) and exclusion criteria (qualitative studies taking a ‘little q’ approach; not relating to cancer; focus only on family/carer perspectives; not relating to changed facial appearance; studies of non-facial appearance).

(Qualitative AND ("grounded theory" OR "discourse analysis" OR "interpretative phenomenological analysis" OR "interview*" OR "focus group*" OR “case stud*” OR “mixed method*” OR "thematic analysis" OR ethnograph* OR “narrative analysis” OR phenomenolog* OR psychological OR psychosocial OR “content analysis”))

AND

(Disfigure* OR deform* OR differen* OR surgery OR trauma OR resect*)

AND

(Face OR facial OR head OR craniofacial OR oral OR mouth OR orofacial OR orbitofacial OR nasal OR nose)

AND

(Cancer* OR tumour OR tumor OR neoplasm OR carcinoma OR malignan*)

**Classifications of quality appraisal**

| Category | Abbreviation | Definition |
| --- | --- | --- |
| Key Paper | KP | Methodologically sound and highly relevant. |
| Satisfactory | SAT | Methodologically sound |
| Questionable | Q | Some methodological issues, included in review but interpreted with caution |
| Fatal Flaw | FF | Fatal flaw in methodology, included in review but with significant caution |

**Quality appraisal tool for qualitative studies**

This checklist is based on:
1) Spencer, L., Ritchie, J., Lewis, J., & Dillon, L. (2003). *Quality in qualitative evaluation: a framework for assessing research evidence.* London: National Centre for Social Research/Cabinet Office.

Available from:
<http://dera.ioe.ac.uk/21069/2/a-quality-framework-tcm6-38740.pdf>

2) Critical Appraisal Skills Programme (CASP). (2013). *10 Questions to help you make sense of qualitative research.*

Available from:
http://media.wix.com/ugd/dded87_29c5b002d99342f788c6ac670e49f274.pdf

**Screening Questions**

1. Was there a clear statement of the aims of the research?

Clear 🞏
Unclear 🞏
Not sure 🞏
Comment:

2. Is a qualitative methodology appropriate?

Clear 🞏
Unclear 🞏
Not sure 🞏
Comment:

Is it worth continuing?

Yes 🞏 If yes, continue to next section.
No 🞏 If no, why not?

**Research design:**

Appropriate 🞏

Inappropriate 🞏

Unsure 🞏

Comment:

**3.** Was the design appropriate to address the research aims?
- is the design justified?
- are any changes justified?

**Sampling:**

Appropriate 🞏

Inappropriate 🞏

Unsure 🞏

Comment:

1. Was the recruitment strategy appropriate?
   – how were participants selected?
   – explanation for suitability of participants recruited?
   - is the sample representative?

- Did any participants not take part

**Data collection:**

Adequate 🞏
Inadequate 🞏
Not sure 🞏
Comment:

**5.** Were the data collected in a way that addressed the research
issue?
– Are all aspects of data collection clear, explicit and theoretically justified? Think about: setting, means, procedure (including interview schedule), modifications to methodology, form of data, saturation of data.

Defensible 🞏

Not defensible 🞏

Unsure 🞏

Comment:

**Reflexivity and validity:**

**6. Has the relationship between researcher and participants been adequately considered?**
– Critical examination of researcher’ role, bias and influence on data throughout the entire research process, including any changes?

**6.1 Is the context clearly described?**
- Clear definition of participants and settings, observations from a variety of circumstances, consideration of context bias.

Clear 🞏
Unclear 🞏
Not sure 🞏
Comment:

Defensible 🞏

Not defensible 🞏

Unsure 🞏

Comment:

**6.2 Were the methods reliable?**
- Multiple methods of data collection, triangulation?
- Do the methods do what they claimed to investigate?

Defensible 🞏

Not defensible 🞏

Unsure 🞏

Comment:

**Ethical Issues:**

**7. Have ethical issues been taken into consideration?**– Enough detail to convey that ethical standards have been maintained, discussion of issues raided by the research, evidence of Ethics Committee approval?

**Data Analysis:**

Adequate 🞏
Inadequate 🞏
Not sure 🞏
Comment:

**8. Was the data analysis sufficiently rigorous?**
– In-depth description of analysis process, clear desctiption of how themes are derived from data, explanation of why selected data how been chosen for presentation, sufficient data presented to verify themes, are contradictory data accounted for?

**8.1 Are the data ‘rich’?**
- Are the contexts of the data clear? Have different viewpoints been explored? Sufficient demonstration of detail and depth? Comparisons between different data sets?

Adequate 🞏
Inadequate 🞏
Not sure 🞏
Comment:

**Findings:
9. How does the evidence support the findings?**
– Findings should be explicit, coherent and credible (triangulation, independent analysts, respondent validation). Findings should be discussed in relation to evidence. Are interpretations justified and clearly argued? Are alternative explanations offered?

Defensible 🞏

Not defensible 🞏

Unsure 🞏

Comment:

**Value of the research:**

**10. How valuable is the research?**
– Do findings fit with extant literature? Consideration of current practice and policy? Further research identified? Has the research question been answered? How do the findings fit with existing knowledge?

Defensible 🞏

Not defensible 🞏

Unsure 🞏

Comment:

**Summary of agreed quality appraisal**

|  | 1. Was there a clear statement of the aims of the research? | 2. Is a qualitative methodology appropriate? | 3. Was the research design appropriate to address the aims of the research? | 4. Was the recruitment strategy appropriate to the aims of the research? | 5. Were the data collected in a way that addressed the research issue? | 6. Has the relationship between researcher and participants been adequately considered? | 6.1. Is the context clearly described? | 6.2. Were the methods reliable? | 7. Have ethical issues been taken into consideration? | 8. Was the data analysis sufficiently rigorous? | 8.1. Are the data 'rich'? | 9. How does the evidence support the findings? | 10. How valuable is the research? | QUALITY |
| --- | --- | --- | --- | --- | --- | --- | --- | --- | --- | --- | --- | --- | --- | --- |
| Furness et al. (2006) | ✔ | ✔ | ✔ GT to explain experience | ✔wide range of recruitment avenues described | ✔description modifying data collection to suit participant needs | Not defensible | ✔adequate description and rationale for heterogeneity of data | ✔Focus groups, interviews and surveys. Independent data audit | Not defensible | ✔step by step description given, sufficient raw data included | ✔good description of contexts from diverse viewpoints | ✔  findings explicit, fits model, could be more coherent | ✔discussion of implications for practice, further research | Satisfactory |
| Konradsen et al. (2009) | ✔ | ✔ | ✔Use of GT to generate a model | ✔homogenous sample of participants | ✔Individual interviews and recorded interactions were analysed until saturation | Not defensible | ✔ | ✔  Research team discussed findings, original data checked | ✔Good description of how ethical issues were addressed | ✔Step by step description given, sufficient raw data included | ✔context given to data and themes, contrasting views expressed | ✔ | ✔good discussion of clinical and theory implications | Key paper |
| Speraw (2009) | ✔ | ✔ | ✔case study to explore the concept of personhood | ✔case study drawn from a larger sample | ✔good account of data collection and detail of interview | ✔field notes were taken after interview and incorporated into analysis | ✔good detail of the context around the case | ✔interview continued until saturation | ✔detailed description of ethical issues at each stage | ✔good description of methods and use of group to verify coding | ✔ rich raw data excerpts, contextual details | ✔findings related to range of theories | Unsure – n=1 means no generalisability | Satisfactory |
| Turpin et al. (2009) | ✔ | ✔aim to explore personal meaning of experience | ✔rationale provided for design | Unsure - insufficient detail of how the 11 participants were recruited | ✔ | Not defensible | ✔useful context re: participants' backgrounds | ✔interviews and repertory grids used | Not defensible | ✔ | ✔ | ✔ | ✔Theory and clinical implications considered | Key paper |
| Van Doorneet al. (2009) | ✔ | ✔aim to understand ppt experience | ✔design suitable for aims | Unsure - limited detail on how final sample was selected | Partially defined – insufficient detail on interviews | Not defensible | Unclear | Inadequate - no description of triangulation | Not defensible | Inadequate - no description of analytical process | Inadequate - insufficient depth of reporting | Inadequate - almost no comparison to literature | Unsure – minimal mention of clinical implications | Questionable |
| Bonanno & Choi (2010) | ✔ | ✔outlined and specific to interactions with secondary groups | ✔Qualitative methods appropriate given under-theorised area | Unsure - limited detail of strategy | ✔sufficient detail provided on methodology | Not defensible | Unclear - little detail | Inadequate - insufficient detail provided on procedure of GT | Not defensible | Unsure - little detail on how themes and patterns have been identified | ✔ | ✔good discussion of interpretation and findings in relation to literature | ✔ | Questionable |
| Konradsen et al. 2012. | ✔ | ✔ | ✔ | ✔follow-up of previous study, in order to explore adjustment | ✔ | Not defensible | ✔good background of previous study provided | ✔sufficient detail of interview procedure | ✔thorough account | ✔step by step description given, sufficient raw data included | ✔context of data well described | ✔ | ✔ | Satisfactory |
| Bonano & Esmaeli (2012) | ✔ | ✔aim to explain experience of interaction | ✔semi-structured interviews | ✔theoretical sampling in line with GT until saturation | ✔ | Not defensible | ✔ | ✔ | Not defensible | ✔description of analysis process given, sufficient raw data | ✔ | Unsure - discussion in relation to literature is limited | ✔ | Satisfactory |
| McGarvey et al. (2014) | ✔ | ✔ | Unsure - no specific qualitative methodology named | ✔ | Partially defined -some interviews very short (3 minutes) | Not defensible | ✔ | Unsure - median length of interview was 10 minutes - short | ✔ | ✔adequate description of analysis, reviewed by 2nd researcher | Unsure- limited data collected, but various views dicussed | ✔ | ✔explicit discussion of clinical implications | Satisfactory |
| Costa et al. (2014) | ✔ | ✔seeking to understand experience | ✔ | ✔sample limited to population covered by aims | ✔semi-structured interview focusing on aspects of facial changed facial appearance | ✔Researchers background considered as well as wider cultural and political issues | ✔sufficient detail of background | ✔good detail provided which ensures replicability | ✔ | ✔thorough account of GT, raw data included | ✔ | ✔ | ✔linking to literature and clinical implications | Satisfactory |
| Henry et al. (2014) | ✔ | ✔aim to explore lived experience | ✔good use of interviews using IPA | ✔use of maximum variation sampling | ✔good description of interview methodology | ✔helpful account of reflexive factors provided | ✔ | ✔ | Unsure - ethics approval gained but no further description | ✔ | ✔ | ✔ | ✔ | Key Paper |
| Nayak et al. (2016) | ✔ - in abstract only though | ✔mixed methods approach used | Unsure - semi-structured interviews chosen but rationale for design not stated | Unsure - little detail of purposive sampling, no rationale for inclusion of women only | Partially defined - no detail provided beyond "semi-structured interview on body image" | Not defensible | Not defensible - characteristics of participants not described | Unsure - triangulation is mentioned but no detail is provided | ✔ | Unsure - some information about use of Colaizzi process, but not detailed enough | Inadequate | Inadequate - not in clear English, limited critiqueof literature | Inadequate | Fatal Flaw |
| Lee et al. (2016) | ✔ | ✔ | ✔semi-structured interviews to identify patient experience | ✔purposive sampling of eligible participants at a large cancer centre | ✔useful inclusion of interview schedule, details of modifications | Not defensible | ✔good detail of participant characteristics | Unsure - not specified if triangulation/ multiple coders were used | ✔ | Unsure - details provided of analysis process but no raw data included | Unsure - some rich description of data, but raw data not included | ✔coherent and relevant findings and evidence discussed | ✔contributing towards an outcome measure | Satisfactory |

**Summary table of second and third order constructs**

| Category | 2nd order construct | Summary definition | Papers included |
| --- | --- | --- | --- |
| Changes to sense of self | Ruptured sense of self | Sense of self as an individual person with a unique role in the world is damaged by the changed facial appearance, and there is a gradual process of rebuilding a new self as congruent with previous identity, as a survivor, and developing new strengths | 11, 4 |
|  | Destruction of valued roles |  | 4 |
|  | Reconstructing own identity |  | 10, 11, 4 |
|  | Striving to develop as a person |  | 11, 10 |
|  | Wanting a better future |  | 10, 11 |
|  | Adjustment is a gradual process |  | 10, 5 |
|  | Striving to regain sense of self |  | 11, 4 |
|  | Valued new identity as a survivor |  | 4, 3 |
| Self to self-relating | Negative body image | View of altered self as negative and incongruent, leading to more awareness and attempts at avoidance of feelings about appearance | 9, 4, 11 |
|  | Anger and shame towards changed facial appearance |  | 3 |
|  | Noticing own emotions and responses more |  | 7 |
|  | Avoiding own emotions and thoughts |  | 4 |
|  | I'm unattractive, ugly |  | 4, 11, 9 |
|  | I'm inadequate |  | 11, 4 |
|  | Shock in looking at self |  | 11, 10 |
|  | I'm different |  | 9, 11, |
|  | I'm not me |  | 4 |
| The self in the world | Being conspicuous, garnering attention | The feeling of drawing a lot of attention by virtue of being different | 11, 4, 6, 7, 8 |
| Others' reactions | Making others uncomfortable | Others’ negative reactions and the amount of attention given to the changed facial appearance in interactions plays an important role | 3, 10, 11, 6, 8 |
|  | Provoking pity, sadness and sympathy |  | 11, 10 |
|  | Provoking shock and disgust |  | 11, 10 |
|  | Stigma |  | 10, 8,6 |
|  | Provoking staring, comments and questions |  | 6, 8, 11, |
|  | Being avoided |  | 4, 3, 10 |
|  | I'm inferior compared to society's standards |  | 7, 10, 8, 6, 11 |
|  | Feeling judged by others |  | 11,5 |
| Acceptance by others | Interactional integration of changed facial appearance | Changed facial appearance becomes integrated into social interactions when the individual and others break the silence; it becomes more normal. The closeness of the relationship with others impacts on how interactions are experienced. | 11 |
|  | Close relationships, acquaintances, strangers |  | 4 |
| Relationship with the disease | Emotional journey - Shock, denial, fear, relief, worry | Individuals go through a wide range of emotions and practical problems over the course of the disease; the ongoing changed facial appearance often serves as a reminder of the disease. | 10, 13, |
|  | Changed facial appearance as a reminder of disease |  | 11, |
|  | Practical problems |  | 13 |
|  | Attempts to reduce likelihood of disease |  | 13 |
| Survival is paramount | Changed facial appearance is a luxury issue | Surviving is held as more important than being disfigured. | 2, 5, 12, 10 |
|  | Changed facial appearance is the trade-off for survival |  | 10, 11, 13, |
| Coping | Social avoidance | Individuals' attempts to cope with changed facial appearance and appear "normal" by avoiding social situations and concealing the changed facial appearance. Personality, age, gender, life events and religion all play a role in how people try to cope. | 9, 7, 11, 10 |
|  | Concealment |  | 4, 10, 11, 13, 9 |
|  | Role of age |  | 12, 13, 4 |
|  | Role of gender |  | 9, 11 |
|  | Role of personality |  | 11, 1 |
|  | Role of past and concurrent events |  | 11, 1, |
|  | Role of spirituality and religion |  | 10 |
| The care team | Changed facial appearance is silenced | The role of the care team in managing changed facial appearance is important to individuals, especially being treated in a humane way which acknowledges the changed facial appearance but does not discriminate against it. | 2, 3 |
|  | Being treated as human |  |  |
|  | How changed facial appearance is dealt with is important |  | 13, 11, 3 |

**Example of a conceptual map.** Conceptual maps were drawn from each paper to aid the interpretive process. Each map contained the second order constructs identified in the paper, and illustrates how constructs fit with the original authors’ findings. This conceptual map from the Costa et al. (2014) study is presented as an example.

Relationship with disease

Relationship with disease

Comparisons to old self?

The patient’s new life experience

Seeing changed facial appearance as the price for survival

Reactions of others

Reconstructing a new identity

Facing the disease and coping with changed facial appearance

Discovery of the cancer
